# Cardiac output and arteriovenous oxygen difference contribute to lower peak oxygen uptake in patients with fibromyalgia

**DOI:** 10.1186/s12891-023-06589-2

**Published:** 2023-07-01

**Authors:** Taneli Lehto, Teemu Zetterman, Ritva Markkula, Jari Arokoski, Heikki Tikkanen, Eija Kalso, Juha E. Peltonen

**Affiliations:** 1 Department of Sports and Exercise Medicine, Clinicum, University of Helsinki, Mäkelänkatu 47, Urhea-Hall, 00550 Helsinki, Finland; 2 Department of Physical and Rehabilitation Medicine, Helsinki University Hospital and Helsinki University, Helsinki, Finland; 3 Department of Anaesthesiology, Intensive Care and Pain Medicine, Pain Clinic, Helsinki University and Helsinki University Hospital, Helsinki, Finland; 4 City of Vantaa Health Centre, Vantaa, Finland; 5 Department of General Practice and Primary Health Care, University of Helsinki, Helsinki, Finland; 6 Sports and Exercise Medicine, Institute of Biomedicine, University of Eastern Finland, Kuopio, Finland; 7 SLEEPWELL Research Programme, Faculty of Medicine, University of Helsinki, Helsinki, Finland; 8 Department of Pharmacology, Faculty of Medicine, University of Helsinki, Helsinki, Finland; 9 Foundation for Sports and Exercise Medicine, Helsinki Sports and Exercise Medicine Clinic, Helsinki, Finland

**Keywords:** Cardiopulmonary exercise test, Impedance cardiography, Leisure-time physical activity, Ventilatory threshold, Oxygen cost

## Abstract

**Background:**

Patients with fibromyalgia (FM) exhibit low peak oxygen uptake ($\dot{\text{V}}$O_2peak_). We aimed to detect the contribution of cardiac output to ($\dot{\text{Q}}$) and arteriovenous oxygen difference $[\text{C}(\text{a-v})\text{O}_{2}]$ to $\dot{\text{V}}\text{O}_{2}$ from rest to peak exercise in patients with FM.

**Methods:**

Thirty-five women with FM, aged 23 to 65 years, and 23 healthy controls performed a step incremental cycle ergometer test until volitional fatigue. Alveolar gas exchange and pulmonary ventilation were measured breath-by-breath and adjusted for fat-free body mass (FFM) where appropriate. $\dot{\text{Q}}$ (impedance cardiography) was monitored. $\text{C}(\text{a-v})\text{O}_{2}$ was calculated using Fick’s equation. Linear regression slopes for oxygen cost (∆$\dot{\text{V}}$O_2_/∆work rate) and $\dot{\text{Q}}$ to $\text{V}$O_2_ (∆$\dot{\text{Q}}$/∆$\dot{\text{V}}$O_2_) were calculated. Normally distributed data were reported as mean ± SD and non-normal data as median [interquartile range].

**Results:**

$\dot{\text{V}}$O_2peak_ was lower in FM patients than in controls (22.2 ± 5.1 vs. 31.1 ± 7.9 mL∙min^−1^∙kg^−1^, *P* < 0.001; 35.7 ± 7.1 vs. 44.0 ± 8.6 mL∙min^−1^∙kg FFM^−1^, *P* < 0.001). $\dot{\text{Q}}$ and C(a-v)O_2_ were similar between groups at submaximal work rates, but peak $\dot{\text{Q}}$ (14.17 [13.34–16.03] vs. 16.06 [15.24–16.99] L∙min^−1^, *P* = 0.005) and C(a-v)O_2_ (11.6 ± 2.7 vs. 13.3 ± 3.1 mL O_2_∙100 mL blood^−1^, *P* = 0.031) were lower in the FM group. No significant group differences emerged in ∆$\dot{\text{V}}$O_2_/∆work rate (11.1 vs. 10.8 mL∙min^−1^∙W^−1^, *P* = 0.248) or ∆$\dot{\text{Q}}$/∆$\dot{\text{V}}$O_2_ (6.58 vs. 5.75, *P* = 0.122) slopes.

**Conclusions:**

Both $\dot{\text{Q}}$ and C(a-v)O_2_ contribute to lower $\dot{\text{V}}$O_2peak_ in FM. The exercise responses were normal and not suggestive of a muscle metabolism pathology.

**Trial registration:**

ClinicalTrials.gov, NCT03300635. Registered 3 October 2017—Retrospectively registered. https://clinicaltrials.gov/ct2/show/NCT03300635.

## Background

The key symptoms of fibromyalgia (FM) include persistent, widespread pain, disturbed sleep, fatigue, and cognitive and mood disturbances [1]. The exact pathophysiology of FM remains unknown. Central sensitization and defects in endogenous pain inhibition are now recognized, but peripheral factors may be equally pertinent [1]. The muscle in FM has been investigated since the 1980s [2], but compelling evidence of altered muscle function in FM is still lacking.

Aerobic and strengthening exercise are strongly recommended in the multimodal management of FM [3], although exercise-induced worsening of symptoms is commonly reported [4]. Nevertheless, physiological adaptations to endurance [5] and resistance [6] exercise are comparable to those of healthy controls. Patients with FM have low peak oxygen uptake ($\dot{\text{V}}$O_2peak_) [7] and $\dot{\text{V}}$O_2peak_ is associated with pain severity [8] in FM. Physical inactivity [9] is a conceivable explanation for low $\dot{\text{V}}$O_2peak_, but it is not known which of its contributing factors, cardiac output ($\dot{\text{Q}}$) or arteriovenous oxygen difference (C(a-v)O_2_), is limiting aerobic capacity in FM. Although FM per se does not seem to increase mortality [10], low cardiorespiratory fitness is a risk factor for all-cause mortality and morbidity [11] and is therefore a relevant health issue.

Mitochondrial pathology, also suggested to be a part of the pathophysiology of FM [12–16], would be an intriguing explanation tying together exercise intolerance and the muscle symptoms of FM. The reason for these putative mitochondrial alterations is not known, and most of the studies do not account for physical activity. However, a genetic polymorphism in mitochondrial DNA, resulting in decreased oxidative phosphorylation, has been suggested to associate with FM [17]. Gerdle et al. [16] found higher pyruvate and lower adenosine triphosphate (ATP) and phosphocreatine (PCr) concentrations in the muscles of FM patients, which may reflect decreased cellular respiration in the mitochondria.

Altogether, FM symptoms share similarities with those of mitochondrial myopathies (MM) [15]. MM can be investigated with the cardiopulmonary exercise test (CPET) [18]. CPET findings in MM may include low $\dot{\text{V}}$O_2peak,_ early anaerobic threshold, high respiratory exchange ratio (RER), high resting lactate, high peak minute ventilation to oxygen uptake ratio ($\dot{\text{V}}$ _E_/$\dot{\text{V}}$O_2_), steep heart rate (HR) to oxygen uptake ($\dot{\text{V}}$O_2_) slope (ΔHR/Δ$\dot{\text{V}}$O_2_), and low C(a-v)O_2_, which reflects muscle oxygen extraction [18, 19]. Taivassalo et al*.* [19] found steep $\dot{\text{Q}}$ to $\dot{\text{V}}$O_2_ slopes (Δ$\dot{\text{Q}}$/Δ$\dot{\text{V}}$O_2_) in MM patients. A recent study demonstrated steeper $\dot{\text{V}}$O_2_ to work rate (P) slopes (Δ$\dot{\text{V}}$O_2_/ΔP) in patients with different metabolic (including mitochondrial) myopathies as well as ‘non-metabolic myalgia’ compared with controls [20]. To our knowledge, these slopes have not been studied in FM before.

We hypothesized that a possible pathology in muscle metabolism in patients with FM would result in altered exercise responses in a CPET and that pain intensity would affect exercise capacity. More precisely, if mitochondrial oxygen demand was decreased due to deficits in the cellular respiration pathways or simply due to lower muscle mitochondrial density, this would result in lower oxygen extraction and hence lower C(a-v)O_2_ and $\dot{\text{V}}$O_2peak_ as observed in MM [19]. Our primary objectives were to determine the contributing factors to $\dot{\text{V}}$O_2_, to compare the Δ$\dot{\text{Q}}$/Δ$\dot{\text{V}}$O_2_ and Δ$\dot{\text{V}}$O_2_/ΔP slopes between FM patients and controls, and to explore other exercise responses, including ventilatory thresholds (VTs), stroke volume (SV), systemic vascular resistance (SVR), and ventilatory efficacy (Δ$\dot{\text{V}}$ _E_/Δ$\dot{\text{V}}$CO_2_, where $\dot{\text{V}}$ _E_ is pulmonary ventilation and $\dot{\text{V}}$CO_2_ is carbon dioxide production), among others. We expected to see 1) low $\dot{\text{V}}$O_2peak_ and C(a-v)O_2,_ 2) low VTs, 3) steep Δ$\dot{\text{Q}}$/Δ$\dot{\text{V}}$O_2_ and Δ$\dot{\text{V}}$O_2_/ΔP slopes, and 4) normal cardiac and pulmonary function in patients with FM. The secondary aim was to explore the relations between self-reported leisure-time physical activity (LTPA), disease severity, pain ratings, psychological factors, and exercise capacity.

The work presented here is part of a larger study; Metabolism, Muscle Function, and Psychological Factors in Fibromyalgia, where the participants also underwent an electromyography study and an oral glucose tolerance test.

## Methods

### Study population

**Fig. 1:**
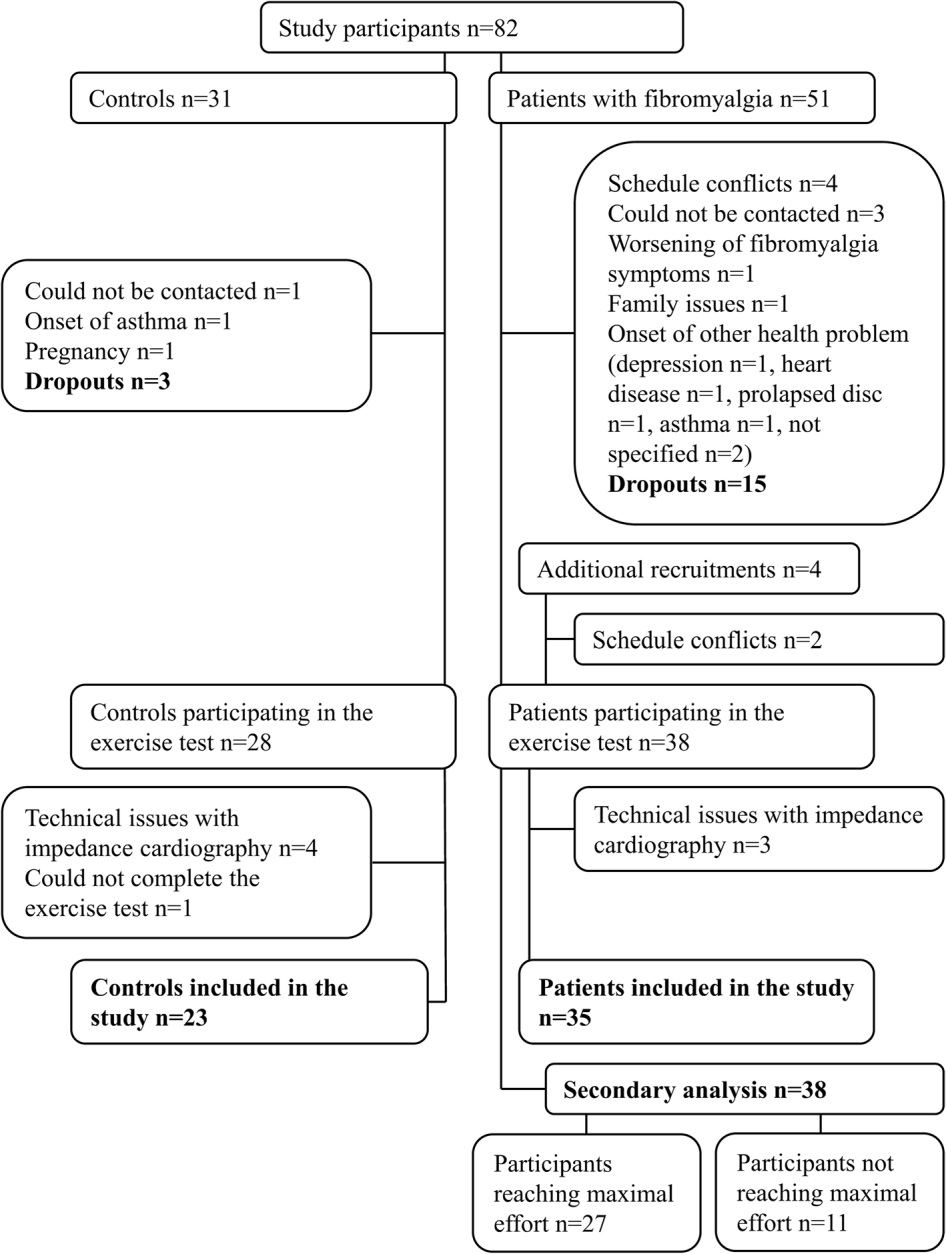
Flowchart of participant recruitment

In total, 38 women with FM and 28 age-matched healthy female controls participated in the exercise test. Of these participants, 35 women with FM, aged 23 to 65 years, and 23 controls completed the test without any technical issues in data recording and were included in the study. The secondary analysis, aiming to identify factors affecting exercise effort, included all 38 women with FM (Fig. 1). The initial recruitment process and exclusion criteria have previously been described [21]. Briefly, the American College of Rheumatology (ACR) 1990 Criteria for Fibromyalgia [22] were used as the inclusion criteria for the FM group. One of the researchers (TZ) performed a clinical examination on patients. Most of the patients were recruited from primary healthcare and from the Helsinki University Central Hospital outpatient clinics. The controls were recruited from the staff of the above-mentioned healthcare units and a local home economic organization (Uudenmaan Martat ry).

### Questionnaires

The participants reported the frequency and duration of their total LTPA and activity at different intensities (light, moderate, heavy). We then combined moderate and heavy physical activity (moderate to heavy) for the analyses, as the volumes of heavy LTPA were low. Other background data were collected utilizing questionnaires completed in the previous phase of the study [21]. These consisted of Finn-FIQ (Finnish version of the Fibromyalgia Impact Questionnaire) [23], PSS (Perceived Stress Scale) [24], STAI (State-Trait Anxiety Inventory) [25], PCS (Pain Catastrophizing Scale) [26], and ACR 2016 Criteria for Fibromyalgia (consisting of Widespread Pain Index [WPI] and Symptom Severity [SS]) questionnaires [27]. The STAI questionnaire comprises two parts: STAI-state, measuring current anxiety, and STAI-trait, measuring anxiety as a trait. In PSS, the timespan is the previous month. The delay between completing the questionnaires and the laboratory visit was long (median 5 months), and we therefore decided to omit STAI-state and PSS. PCS is validated for pain populations and FIQ for FM populations and are therefore not reported for the control group.

### Study protocol

The study protocol is largely adopted from previous studies performed in our laboratory [28, 29]. All measurements (excluding the above-mentioned questionnaires) were performed on a single visit between January 2016 and April 2019.

The participants arrived at the laboratory 2–3 h after a meal (breakfast or lunch). The visit consisted of pre-exercise measurements and a CPET. We measured the participants’ weight, height, and waist-to-hip ratio and calculated the body mass index (BMI). Body composition (*e.g.* fat-free body mass (FFM)) was analyzed using a bioimpedance device (InBody 720; Biospace Co., Ltd., Seoul, South Korea). In women, the InBody device yields roughly 8% higher FFM results compared with dual-energy x-ray absorptiometry [30]. Pre-exercise measurements included also a 12-lead ECG, blood pressure, and flow-volume spirometry (Medikro Spiro 2000; Medikro Oy, Kuopio, Finland). A physician evaluated the participants’ suitability for the exercise test. The CPET was performed on a cycle ergometer (Monark Ergomedic 839E; Monark Exercise AB, Vansbro, Sweden). The step incremental protocol was preceded by a 5-min rest while the subjects sat relaxed on the ergometer followed by a 5-min unloaded cycling (equivalent to ~ 6 W). Incremental exercise (25 W every 3 min) was then initiated, and the subjects continued exercising until volitional fatigue. The participants reported their rate of perceived exertion (RPE) using the Borg scale [31] (range 6 to 20) at the end of each work rate (P). They reported their sensation of pain at rest and after exercise using the numeric rating scale (NRS) (0 to 10).

### Lactate and pyruvate concentrations

We collected blood samples at rest and immediately after exercise. For the pyruvate samples, we drew 1 mL of venous blood into EDTA tubes (Bd Vacutainer K2E 5.4 mg Bd-Plymouth, UK). Then, within 1 min, we pipetted 0.5 mL of blood into two pre-chilled tubes containing 1 mL of 8% perchloric acid each. We cooled the perchloric acid tubes by placing them into a container with cold gel packs for 5 min and then centrifugated them for 10 min at 4 °C and 1500 G. We pipetted the resulting supernatant into one perchloric acid tube. For the lactate samples, we drew 0.5 mL of venous blood into fluoride oxalate tubes (Vacutest NaF + K2OX, Vacutest Rima, Italy), which we then centrifugated for 10 min at 3000 rpm. Both samples were next placed in a freezer at -20.5 °C for a maximum of 3 days and then moved in dry ice to the Helsinki University Hospital Laboratory (HUSLAB) for analysis. The pyruvate samples were analyzed enzymatically by photometry, and the lactate samples were analyzed photometrically. We calculated lactate-to-pyruvate (L/P) ratios for each participant.

### Cardiorespiratory measurements

We measured breath-by-breath ventilation by a low-resistance turbine (Triple V; Jaeger Mijnhardt, Bunnik, the Netherlands) during the exercise test. Expired and inspired gases were sampled continuously at the mouth and analyzed for concentrations of O_2_, CO_2_, N_2_, and Ar by mass spectrometry (AMIS 2000; Innovision A/S, Odense, Denmark) after calibration with precisely analyzed gas mixtures. Breath-by-breath respiratory data were collected as raw data, transferred to a computer to determine gas delays for each breath. The concentrations were aligned with the volume data and the profiles of each breath were built. Breath-by-breath alveolar gas exchange was then calculated with the AMIS algorithms, and the data were interpolated to obtain second-by-second values. $\dot{\text{V}}$O_2peak_ was determined as the highest value of a 60 s moving averaging interval. We analyzed cardiorespiratory responses during exercise at six different time points (*i.e.* work rates): rest, unloaded cycling, 25 W, 50 W, 75 W, and peak exercise, using the mean values of the last 30 s of each step. 75 W was the highest work rate which every participant could reach. VTs were determined as previously reported [32, 33]. Due to the multifaceted terminology, we chose to use the terms ventilatory threshold 1 (VT1) and ventilatory threshold 2 (VT2). We monitored arterial O_2_ saturation (SpO_2_) with fingertip pulse oximetry (Nonin 9600; Nonin Medical, Inc., Plymouth, MA, USA). We evaluated cardiac function with an impedance cardiograph (ICG) device (PhysioFlow; Manatec Biomedical, Paris, France). ICG measures changes in transthoracic impedance during cardiac ejection to calculate SV, which is multiplied by HR to provide an estimate of $\dot{\text{Q}}$. $\dot{\text{Q}}$ determined by ICG during exercise has been validated against the “gold standard”, the direct Fick method [34]. Systolic (SAP) and diastolic (DAP) blood pressures were measured automatically (Tango + ; SunTech Medical, Morrisville, NC, USA) from the brachial artery at rest and at the end of each work rate. We transferred blood pressure values into the ICG device, which calculated mean arterial pressure (MAP) and SVR. The impedance cardiograph data were averaged at 15 s intervals and the average of the last 30 s of each step was used in the analyses. To account for differences in body composition, we calculated indices for $\dot{\text{V}}$O_2_, $\dot{\text{Q}}$ and SV (marked with subscript _i_) by dividing them with FFM, whereas SVR was multiplied with FFM. The evidence for scaling $\dot{\text{V}}$O_2_ to FFM instead of total body weight is robust [35–37]. In addition, research suggests prioritizing FFM over total body weight or body surface area when scaling cardiac function [38, 39]. We defined maximal effort as the inability to maintain a pedalling cadence of 60 rpm and using the age-adjusted RER criteria published by Edvardsen et al. [40].

### Statistical analyses

We assessed the normal distribution of the data with visual inspection and Shapiro–Wilk’s test. Differences between groups (FM and controls) were assessed using unpaired t-tests for normal variables and Mann–Whitney U test for non-normal variables. As not all participants reached maximal effort, we analyzed separately the peak exercise responses after excluding these participants.

We used repeated measures ANOVA, where work rate was a within-subject factor and group a between-subject factor, for the analysis of cardiorespiratory responses during exercise. We then performed a separate MANOVA to further identify the work rates where between-group differences exist.

We analyzed Δ$\dot{\text{V}}$O_2_/ΔP, ΔHR/Δ$\dot{\text{V}}$O_2_, and Δ$\dot{\text{Q}}$/Δ$\dot{\text{V}}$O_2_ slopes with linear regression as previously reported [29]. Group means from five time points (unloaded cycling (~ 6 W), 25 W, 50 W, 75 W, and peak exercise) were included. Resting values were omitted due to the rapid initial increase in oxygen uptake in the transition from rest to unloaded cycling. First, we performed regression analyses, where $\dot{\text{V}}$O_2_ was a dependent variable and work rate an independent variable, for the FM and control groups separately. We then performed another linear regression analysis to evaluate the contribution of FM to the slopes. We created a dummy variable, where the FM group received a value of 1 and the control group a value of 0. The interaction term dummy*independent variable was then included in the model. ΔHR/Δ$\dot{\text{V}}$O_2_, Δ$\dot{\text{Q}}$/Δ$\dot{\text{V}}$O_2,_ and Δ$\dot{\text{V}}$ _E_/Δ$\dot{\text{V}}$CO_2_ slopes were assessed in a similar manner. The range used for the Δ$\dot{\text{V}}$ _E_/Δ$\dot{\text{V}}$CO_2_ slope was from rest until the second ventilatory threshold, after which there is a steep increase in the slope.

We used Spearman correlations to explore the relations between work rate, HR, $\dot{\text{Q}}$, $\dot{\text{V}}$O_2_, and C(a-v)O_2_ at peak exercise, LTPA, and pain.

We conducted a secondary analysis with the FM group. We identified a subgroup of participants who could not reach maximal effort (the ‘submaximal’ group) and they were compared with those who reached maximal effort (the ‘maximal’ group).

All normal data are reported as mean ± SD, non-normal data as median [interquartile range], and categorical data as count (%), unless otherwise stated. Alpha was set to 0.05. The *P* values were not adjusted for multiple comparisons, as increasing type II error was deemed more harmful than reducing type I error. Statistical analyses were conducted using SPSS (IBM SPSS Statistics for Windows, versions 25.0 and 27.0. Armonk, NY, USA).

## Results

### Group demographics

**Fig. 2:**
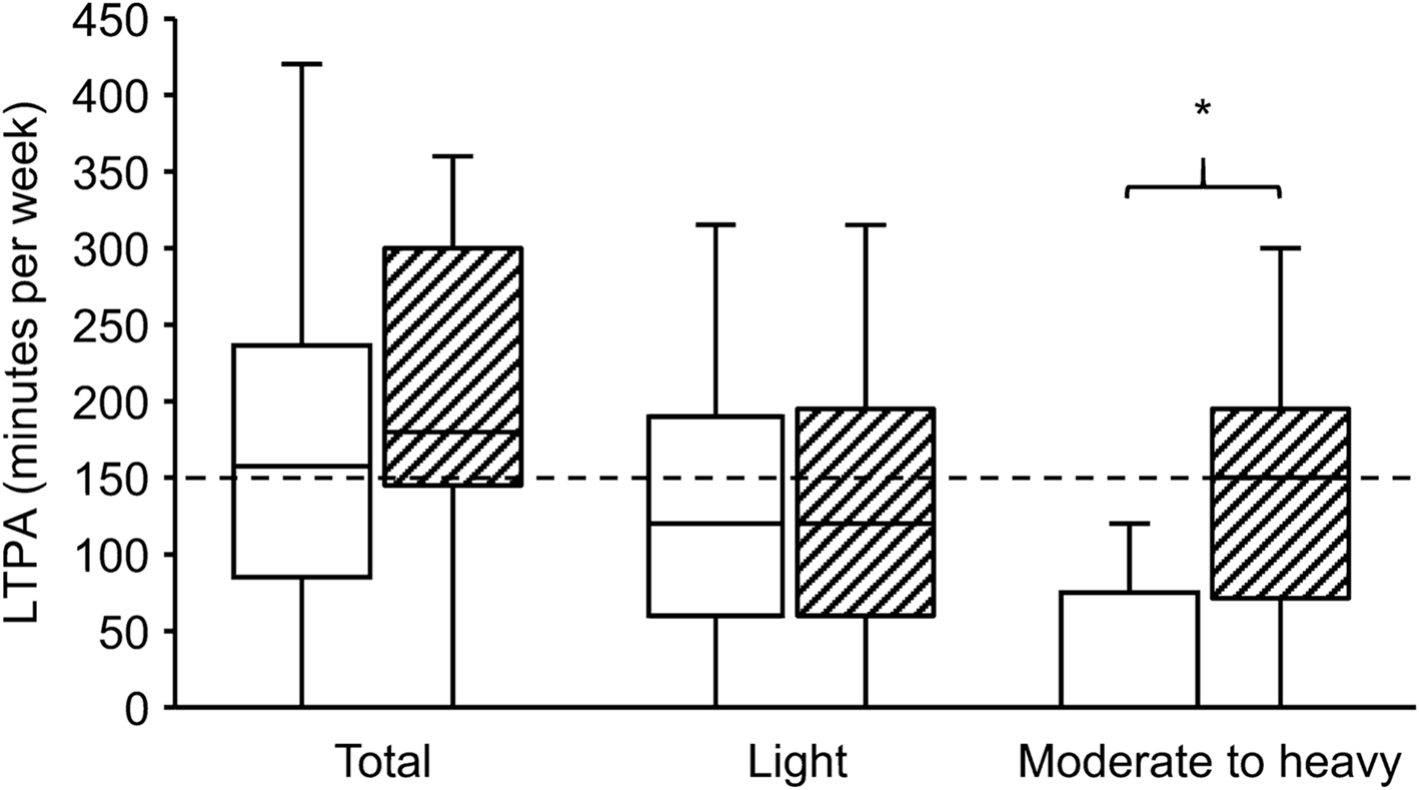
Self-reported leisure-time physical activity. White boxes, fibromyalgia (*n* = 31); shaded boxes, controls (*n* = 23). *, between-group difference significant (*P* < 0.05). Dashed line represents the lower bound of the WHO recommendations for moderate physical activity (see reference 53)

Weight, BMI, body fat percentage, and waist-to-hip ratio were higher and height lower in the FM group, but there was no difference in FFM between the groups. Patients with FM had higher STAI-trait scores, were less likely to be working, had fewer years of education, and had more comorbidities (the three most common being migraine, asthma, and gastroesophageal reflux) than controls. No significant differences were observed in the baseline spirometry values. Background data and spirometry values are shown in Table 1. Self-reported total and light LTPA were similar between groups, but moderate to heavy LTPA was significantly lower in the FM group (Fig. 2). LTPA data were missing for four participants in the FM group.

**Table 1:** Participant data on demography and spirometry

	**Fibromyalgia (*n* = 35)**	**Missing (*n*)**	**Controls (*n* = 23)**	**Missing (*n*)**	***P***
Demographic data					
Age (years)	48.0 [43.1–56.5]		51.1 [39.5–53.7]		0.867^a^
Height (cm)	165 ± 5		168 ± 4		0.032*
Weight (kg)	77.0 ± 15.7		68.5 ± 10.2		0.016*
BMI (kg·m^−2^)	28.4 ± 5.6		24.4 ± 3.4		0.002*
Body fat (%)	38 ± 8		30 ± 8		< 0.001*
Fat-free mass (kg)	46.7 ± 5.2		47.3 ± 3.7		0.605
Waist-to-hip ratio	0.89 [0.82–0.94]	1	0.80 [0.78–0.85]		< 0.001^a^*
Smoking	6 (17)		1 (4)		0.226^b^
Working	22 (65)	1	23 (100)		< 0.001^b^*
Education (years after basic education)	4.7 ± 3.1		8.3 ± 2.1		< 0.001*
Number of other diagnoses	2 [1–3]		0 [0–1]		< 0.001^a^*
ACR 2016 diagnosis	32 (91)		0 (0)		< 0.001^b^*
ACR 2016 WPI	11 [8–15]		1 [0–2]		< 0.001^a^*
ACR 2016 SS	8 [5–9]		2 [1–3]		< 0.001^a^*
ACR 1990 tenderpoint count	16 [13–18]		3 [1–6]		< 0.001^a^*
FIQ	43 ± 15	1	n/a		n/a
PCS	15 [10–23]		n/a		n/a
STAI-trait	46 ± 10	1	28 ± 6	1	< 0.001
Spirometry					
FVC (L)	3.51 ± 0.45		3.68 ± 0.43		0.173
FVC (% ref. value)	96.0 ± 11.4		96.7 ± 11.2		0.827
FEV1 (L)	2.73 ± 0.42		2.92 ± 0.37		0.080
FEV1/FVC	0.78 ± 0.08		0.79 ± 0.06		0.340
PEF (L∙min^−1^)	6.40 ± 0.68		6.77 ± 0.96		0.118

Parametric data expressed as mean ± SD, nonparametric data as median [interquartile range], and categorical data as count (%). *P* values refer to unpaired t-test, except for a, refers to Mann–Whitney U Test, and b, refers to Pearson *Χ*^*2*^ or Fisher’s Exact Test

*ACR* American College of Rheumatology, *WPI* Widespread Pain Index, *SS* Symptom Severity, *FIQ* Fibromyalgia Impact Questionnaire, *PCS* Pain Catastrophizing Scale, *STAI* State-Trait Anxiety Inventory, *FVC* forced vital capacity, *FEV1* forced expiratory volume in one second, *PEF* peak expiratory flow

^*^P < 0.05

^a^Mann-Whitney U Test

^b^Pearson *Χ*^*2*^ or Fisher’s Exact Test

### Baseline heart rate, blood pressure, lactate and pyruvate at rest

HR (85 ± 13 bpm vs. 80 ± 12 bpm, *P* = 0.145), SAP (124 [117–140] mmHg vs. 118 [111–137] mmHg, *P* = 0.112), and DAP (90 ± 8 mmHg vs. 85 ± 9 mmHg, *P* = 0.072) were not significantly different between FM and control groups, whereas mean arterial pressure (100 [96–109] mmHg vs. 96 [91–102] mmHg, *P* = 0.042) was higher in the FM group. No significant differences emerged in resting lactate (0.9 [0.7–1.2] mmol∙L^−1^ vs. 0.9 [0.7–1.2] mmol∙L^−1^, *P* = 0.694), pyruvate (92 [82–98] µmol∙L^−1^ vs. 91 [80–100] µmol∙L^−1^, *P* = 0.920), or L/P ratio (10.6 [8.3–12.9] vs. 10.8 [8.6–13.0], *P* = 0.610) between the groups. Lactate and pyruvate data were missing for four participants in both groups.

### Responses to incremental exercise

**Fig. 3:**
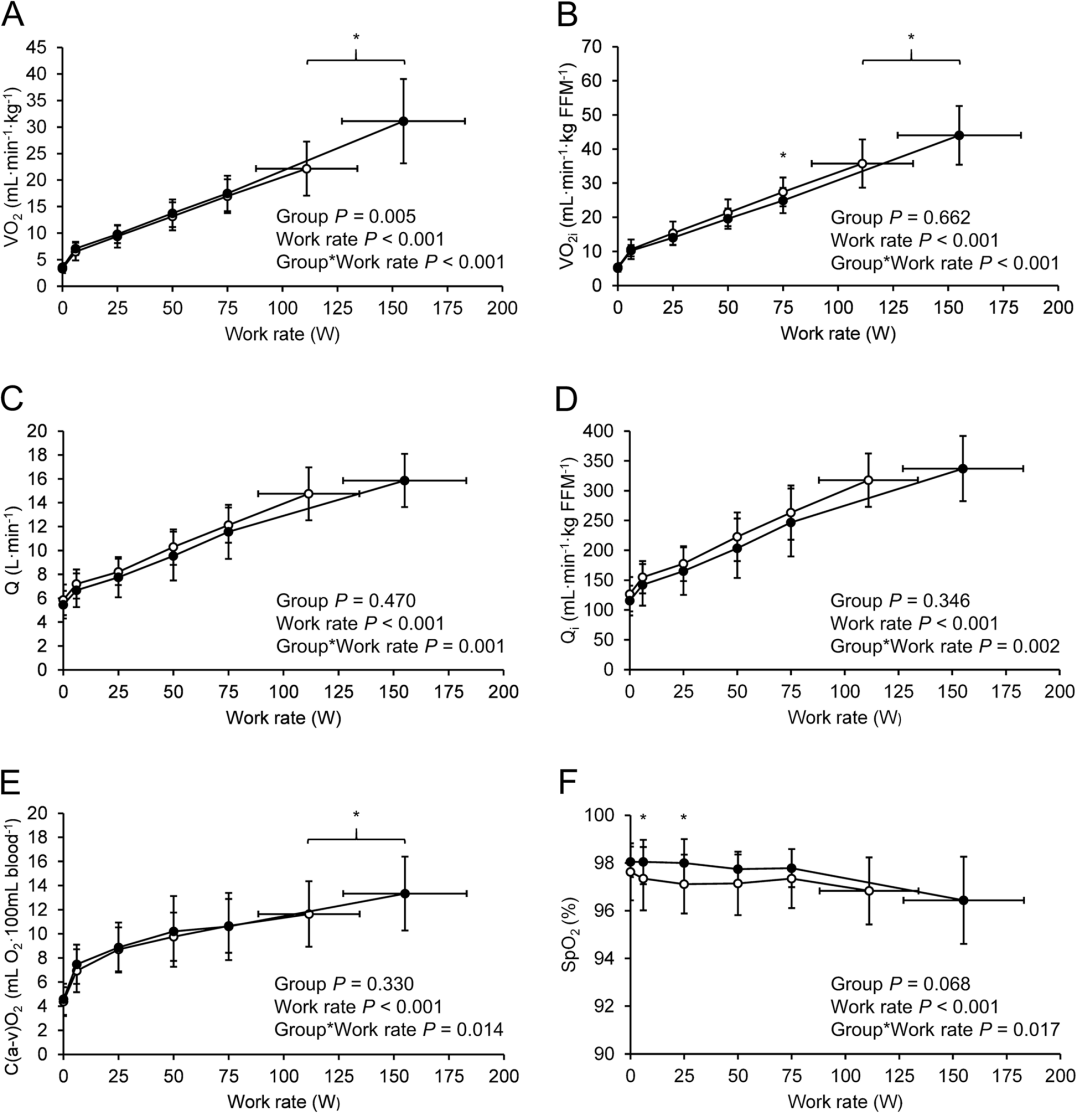
Oxygen uptake (**A**-**B**), cardiac output (**C**-**D**), arteriovenous oxygen difference (**E**), and arterial oxygen saturation (**F**) as a function of work rate. White circles (○), fibromyalgia (*n* = 35); black circles (●), controls (*n* = 23). Values are group means, vertical error bars ± SD. Horizontal error bars represent ± SD of mean peak work rate. *P* values refer to repeated measures ANOVA. *, between-group difference significant (*P* < 0.05) at given work rate

**Fig. 4:**
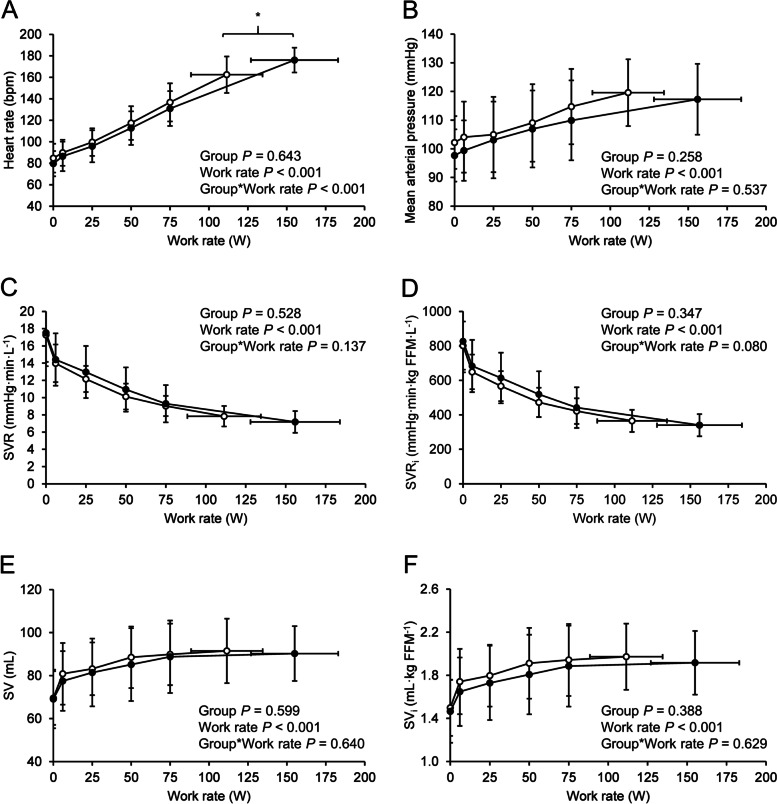
Heart rate (**A**), mean arterial pressure (**B**), systemic vascular resistance (**C**-**D**), and stroke volume (**E**–**F**) as a function of work rate. White circles (○), fibromyalgia (*n* = 35); black circles (●), controls (*n* = 23). Values are group means, vertical error bars ± SD. Horizontal error bars represent ± SD of mean peak work rate. *P* values refer to repeated measures ANOVA. *, between-group difference significant (*P* < 0.05) at given work rate

Figure 3 illustrates the exercise responses for $\dot{\text{V}}$O_2_ and its contributing factors. Significant group*work rate interactions were observed in $\dot{\text{V}}$O_2_, $\dot{\text{V}}$O_2i_, $\dot{\text{Q}}$, $\dot{\text{Q}}$ _i,_ and C(a-v)O_2,_ although the between-group differences were small at submaximal work rates. C(a-v)O_2_ slopes of the two groups were almost identical until peak exercise. SpO_2_ was within normal range throughout the exercise in both groups, but a group*work rate interaction was noted. Other cardiovascular response slopes are shown in Fig. 4. HR in the FM group was lower at peak exercise, and a significant group*work rate interaction was observed. MAP, SV, SV_i_, SVR, and SVR_i_ showed no significant group*work rate interactions.

### Ventilatory thresholds

Participants with FM reached both VT1 and VT2 at lower work rates (51 ± 17 W vs. 65 ± 23 W, *P* = 0.009, and 93 ± 22 W vs. 118 ± 23 W, *P* < 0.001) and lower oxygen consumption (13 ± 4 mL∙min^−1^∙kg^−1^ vs. 16 ± 5 mL∙min^−1^∙kg^−1^, *P* = 0.008, and 20 ± 5 mL∙min^−1^∙kg^−1^ vs. 25 ± 6 mL∙min^−1^∙kg^−1^, *P* < 0.001) than controls. When adjusted for FFM, the difference in oxygen consumption at VT1 was no longer significant (21 ± 5 mL∙min^−1^∙kg FFM^−1^ vs. 23 ± 5 mL∙min^−1^∙kg FFM^−1^, *P* = 0.176), but significance remained at VT2 (32 ± 6 mL∙min^−1^∙kg FFM^−1^ vs. 35 ± 6 mL∙min^−1^∙kg FFM^−1^, *P* = 0.034). $\dot{\text{V}}$O_2_ at VTs as a percentage of $\dot{\text{V}}$O_2peak_ (VT1% and VT2%) was higher in the FM group (60 ± 9% vs. 53 ± 7%, *P* = 0.002, and 88 ± 8% vs. 81 ± 6%, *P* < 0.001). VTs could not be determined for one participant in the FM group, as no clear breakpoints were visible.

### Peak exercise

Peak RER and RPE between the groups were comparable. Altogether 25 participants (71%) in the FM group and 21 (91%) in the control group (Fisher’s exact test, *P* = 0.099) fulfilled the RER criteria for maximal effort. Peak HR (HR_peak_) and HR as a percentage of predicted heart rate were lower and breathing reserve (BR) higher in the FM group. Peak work rate (P_peak_), $\dot{\text{V}}$O_2_, $\dot{\text{V}}$O_2i_, $\dot{\text{V}}$CO_2,_ and $\dot{\text{V}}$_E_ were lower in the FM group**.** PETO_2_ was lower (117 ± 5 mmHg vs. 120 ± 4 mmHg, *P* = 0.020) and PETCO_2_ higher (35 ± 4 mmHg vs. 33 ± 3 mmHg, *P* = 0.014) in the FM group. No significant differences were seen in peak $\dot{\text{V}}$_E_/$\dot{\text{V}}$CO_2_, $\dot{\text{V}}$_E_/$\dot{\text{V}}$O_2_, VD/VT, or SpO_2_ between FM and control groups**.** Peak SAP, DAP, and MAP were similar between groups. Oxygen pulse was slightly lower in the FM group (10.5 ± 2.2 mL∙beat^−1^ vs. 11.7 ± 2.1 mL∙beat^−1^, *P* = 0.028). $\dot{\text{Q}}$ was lower in the FM group but failed to reach statistical significance when adjusted for FFM ($\dot{\text{Q}}$ _i_). Neither SV nor SV_i_ were significantly different between groups at peak exercise. SVR was higher in the FM group, but SVR_i_ failed to reach statistical significance. A significant difference was seen in C(a-v)O_2_ at peak exercise. Peak exercise results for key parameters are shown in Table 2. Peak exercise responses and between group differences remained similar when those not reaching maximal effort were excluded (columns FM_me_ and CTRL_me_ in Table 2).

**Table 2:** Values at peak exercise

	**Fibromyalgia (*n* = 35)**	**Controls (*n* = 23)**	***P***	**FM**_**me**_** (*n* = 25)**	**CTRL**_**me**_** (*n* = 21)**	***P***
Work rate (W)	111 ± 23	155 ± 28	< 0.001*	111 ± 22	153 ± 28	< 0.001*
RPE (Borg)	18 [17–19]	19 [18–19]	0.304^a^	19 [17–20]	19 [18–19]	0.794^a^
$\dot{\text{V}}$O_2_ (L∙min^−1^)	1.66 ± 0.35	2.08 ± 0.44	< 0.001*	1.65 ± 0.32	2.05 ± 0.44	< 0.001*
$\dot{\text{V}}$O_2_ (mL∙min^−1^∙kg^−1^)	22.2 ± 5.1	31.1 ± 7.9	< 0.001*	22.5 ± 5.3	29.9 ± 7.1	< 0.001*
$\dot{\text{V}}$O_2i_ (mL∙min^−1^∙kg FFM^−1^)	35.7 ± 7.1	44.0 ± 8.6	< 0.001*	35.3 ± 6.5	42.9 ± 8.0	< 0.001*
$\dot{\text{V}}$CO_2_ (L∙min^−1^)	1.94 ± 0.42	2.39 ± 0.45	0.001*	1.99 ± 0.42	2.38 ± 0.48	0.005*
$\dot{\text{V}}$_E_ (L∙min^−1^)	67.1 ± 13.9	87.0 ± 17.0	< 0.001*	69.0 ± 12.3	86.5 ± 17.6	< 0.001*
$\dot{\text{V}}$_E_/$\dot{\text{V}}$O_2_	40.8 ± 7.1	42.1 ± 4.3	0.402	42.6 ± 7.3	42.5 ± 4.3	0.956
$\dot{\text{V}}$_E_/$\dot{\text{V}}$CO_2_	34.9 ± 5.2	36.6 ± 3.7	0.159	35.4 ± 5.7	36.5 ± 3.8	0.447
RER	1.14 [1.09–1.21]	1.15 [1.11–1.18]	0.943^a^	1.17 [1.14–1.26]	1.15 [1.12–1.18]	0.127^a^
Breathing reserve (%)	33 ± 0.18	20 ± 0.13	0.004*	30 ± 18	20 ± 14	0.026*
VD/VT	0.14 [0.12–0.17]	0.14 [0.12–0.15]	0.581^a^	0.14 [0.12–0.18]	014 [0.12–0.16]	0.556^a^
SpO_2_ (%)	97 [96–98]	97 [96–98]	0.404^a^	97 [96–98]	97 [96–98]	0.195^a^
Heart rate (bpm)	167 [149–171]	174 [169–187]	0.001^a^*	168 [150–179]	174 [167–184]	0.020^a*^
Heart rate (% of predicted max.)	90 ± 9	97 ± 6	0.001*	91 ± 8	97 ± 5	0.011*
Systolic blood pressure (mmHg)	180 ± 25	183 ± 22	0.676	180 ± 25	184 ± 23	0.625
Diastolic blood pressure (mmHg)	89 ± 9	83 ± 15	0.090	91 ± 10	85 ± 13	0.103
Mean arterial pressure (mmHg)	120 ± 12	117 ± 13	0.347	120 ± 12	118 ± 12	0.462
SV (mL)	87 [82–96]	88 [86–98]	0.541^a^	87 [82–95]	88 [86–99]	0.384^a^
SV_i_ (mL∙kg FFM^−1^)	1.97 ± 0.31	1.92 ± 0.30	0.493	1.97 ± 0.30	1.91 ± 0.30	0.497
$\dot{\text{Q}}$ (L∙min^−1^)	14.2 [13.3–16.0]	16.1 [15.2–17.0]	0.005a*	14.1 [13.4–15.9]	16.1 [15.1–16.9]	0.018^a*^
$\dot{\text{Q}}$_i_ (mL∙kg FFM^−1^)	318 ± 45	337 ± 55	0.148	320 ± 45	332 ± 52	0.416
SVR (mmHg∙min∙L^−1^)	7.84 ± 1.19	7.11 ± 1.27	0.032*	7.82 ± < 1.12	7.24 ± 1.23	0.103
SVR_i_ (mmHg∙min∙kg FFM∙L^−1^)	365 ± 64	337 ± 65	0.107	364 ± 55	344 ± 60	0.243
C(a-v)O_2_ (mL O_2_ ∙100 mL blood^−1^)	11.6 ± 2.7	13.3 ± 3.1	0.031*	11.4 ± 2.7	13.3 ± 3.1	0.031*

Parametric data expressed as mean ± SD and nonparametric data as median [interquartile range]. *P* values refer to unpaired t-test, except for a, refers to Mann–Whitney U test

*FM*_*me*_ maximal effort fibromyalgia group, *CTRL*_*me*_ maximal effort control group, *RPE* rate of perceived exertion, ${\dot{V}}$*O*_*2*_ oxygen uptake, ${\dot{V}}$*CO*_*2*_ carbon dioxide production, ${\dot{V}}$_*E*_ ventilation, *RER* respiratory exchange ratio, *VD/VT* dead space to tidal volume ratio, *SpO*_*2*_ oxygen saturation, *SV* stroke volume, *SV*_*i*_ stroke volume index, ${\dot{Q}}$ cardiac output, ${\dot{Q}}$_*i*_ cardiac output index, *SVR* systemic vascular resistance, *SVR*_*i*_ systemic vascular resistance index, *C(a-v)O*_*2*_ arteriovenous oxygen difference

^*^P < 0.05

^a^Mann Whitney U Test

### Postexercise lactate, pyruvate and L/P ratio

The FM group had lower postexercise lactate concentration (8.1 [6.1–10.0] mmol∙L^−1^ vs. 11.1 [9.1–12.5] mmol∙L^−1^, *P* = 0.003) and L/P ratio (58.6 [44.5–78.6] vs. 71.4 [59.4–88.3], *P* = 0.032), while postexercise pyruvate concentrations were similar (140 [123–155] µmol∙L^−1^ vs. 155 [122–167] µmol∙L^−1^, *P* = 0.184) between groups. Data were missing for four participants in both groups.

### Δ$\dot{\text{V}}$O_2_/ΔP, ΔHR/Δ$\dot{\text{V}}$O_2_, Δ$\dot{\text{Q}}$/Δ$\dot{\text{V}}$O_2_, and Δ$\dot{\text{V}}$E/Δ$\dot{\text{V}}$CO_2_ slopes

**Fig. 5:**
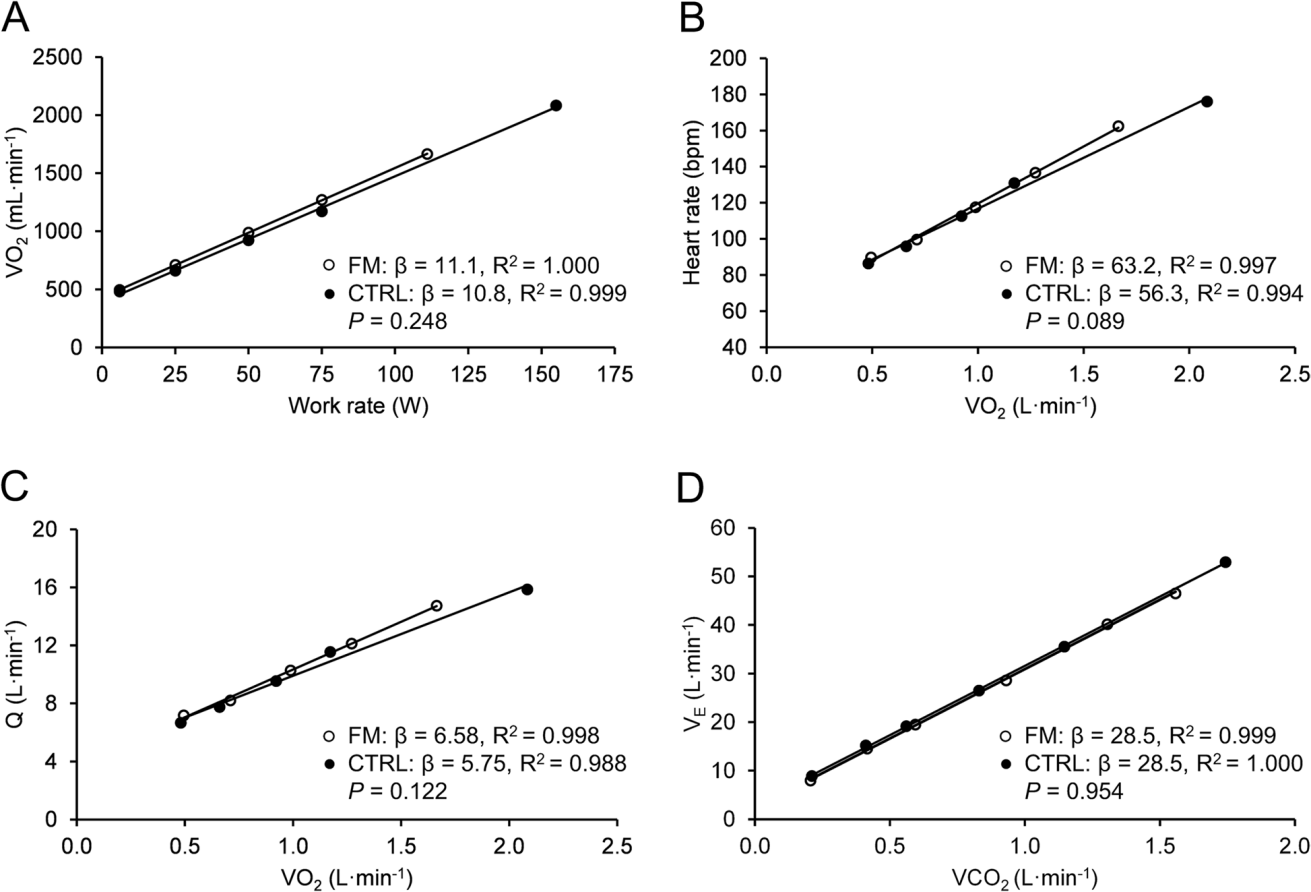
Linear regression slopes for oxygen uptake as a function of work rate (**A**), heart rate (**B**) and cardiac output (**C**) as a function of oxygen uptake, and ventilation as a function of carbon dioxide production (ventilatory efficacy) (**D**). White circles (○), fibromyalgia (*n* = 35, except for panel D, *n* = 34); black circles (●), controls (*n *= 23). *P* values refer to the group*independent variable term in the regression model (see text for more information)

Δ$\dot{\text{V}}$O_2_/ΔP, ΔHR/Δ$\dot{\text{V}}$O_2,_ Δ$\dot{\text{Q}}$/Δ$\dot{\text{V}}$O_2_, and Δ$\dot{\text{V}}$ _E_/Δ$\dot{\text{V}}$CO_2_ slopes were similar between groups, and the FM*independent variable interactions were not significant. Linear regression slopes are shown in Fig. 5.

### Pain

The FM group reported higher pain NRS at rest (3 [2–5] vs. 0 [0–0], *P* < 0.001) and after exercise (5.5 [3–8] vs. 0 [0–1], *P* < 0.001), with a median change of 2 (Wilcoxon Signed-Rank Test, *P* < 0.001). Of the FM patients, 24 (71%) experienced an increase in pain, whereas 8 (24%) reported no change and 2 (6%) a decrease in pain. Data were missing for one participant in the FM group. In the FM group, a negative correlation with baseline pain NRS and $\dot{\text{V}}$O_2peak_ (*ρ* = -0.46, *P* = 0.007) and P_Peak_ (*ρ* = -0.43, *P* = 0.011) was observed. Postexercise pain NRS correlated negatively only with $\dot{\text{V}}$O_2peak_ (*ρ* = -0.40, *P* = 0.018). No significant associations emerged between the change in pain ratings (post–pre) and $\dot{\text{V}}$O_2peak_ (*ρ* = -0.23, *P* = 0.183) or P_peak_ (*ρ* = -0.12, *P* = 0.493). Neither baseline nor postexercise pain NRS correlated with HR_peak_ (*ρ* = -0.31, *P* = 0.079 and *ρ* = -0.16, *P* = 0.355).

### Correlations between LTPA, $\dot{\text{V}}$O_2peak_, P_peak_, HR_peak_, C(a-v)O_2_, $\dot{\text{Q}}$ _peak_ and background data

A positive correlation was observed with $\dot{\text{Q}}$ _peak_ and $\dot{\text{V}}$O_2ipeak_ (*ρ* = 0.30, *P* = 0.022) but not with $\dot{\text{V}}$O_2peak_ (*ρ* = 0.25, *P* = 0.058). Moderate to heavy LTPA correlated with $\dot{\text{V}}$O_2peak_ (*ρ* = 0.60, *P* < 0.001), HR_peak_ (*ρ* = 0.37, *P* = 0.005), and P_peak_ (*ρ* = 0.55, *P* < 0.001) and total LTPA with $\dot{\text{V}}$O_2peak_ (*ρ* = 0.34, *P* < 0.013). However, no correlation was observed between light LTPA and $\dot{\text{V}}$O_2peak_ (*ρ* = 0.03, *P* = 0.839), HR_peak_ (*ρ* = -0.21, *P* = 0.129) or P_Peak_ (*ρ* = -0.02, *P* = 0.906). Peak C(a-v)O_2_ and $\dot{\text{Q}}$ _peak_ correlated only with moderate to heavy LTPA (*ρ* = 0.35, *P* = 0.010 and *ρ* = 0.33, *P* = 0.016). When excluding the controls, a significant correlation remained only with total LTPA and $\dot{\text{V}}$O_2peak_ (*ρ* = 0.37, *P* = 0.040) and moderate to heavy LTPA with $\dot{\text{V}}$O_2ipeak_ (*ρ* = 0.53, *P* = 0.002). FIQ, PCS, STAI-trait, ACR 2016 WPI, or ACR 2016 SS did nor correlate with $\dot{\text{V}}$O_2peak_, P_peak_, HR_peak_ or LTPA in the FM group (data not shown).

### Demographic differences in submaximal and maximal effort FM groups

The submaximal group had higher FIQ and STAI-trait scores. More participants in the submaximal group had a pulmonary diagnosis compared with the maximal group. Altogether eight FM patients had pulmonary comorbidities (asthma, *n* = 7; sleep apnea, *n* = 2; both, *n* = 1). Of the seven patients with concurrent FM and asthma, two reached maximal effort. No significant differences emerged in baseline spirometry between asthmatic and non-asthmatic FM patients or between submaximal and maximal groups (data not shown). Group demographics are shown in Table 3.

**Table 3:** Comparison of submaximal versus maximal effort fibromyalgia groups

	**Submaximal (*n* = 11)**	**Missing (*n*)**	**Maximal (*n* = 27)**	**Missing (*n*)**	***P***
Age (years)	43.3 ± 9.9		48.8 ± 10.6		0.154
Height (cm)	165 ± 5		165 ± 5		0.969
Weight (kg)	78.3 ± 11.9		76.3 ± 17.9		0.741
BMI (kg·m^−2^)	30.6 [23.2–31.5]		26.1 [22.9–33.6]		0.573^a^
Fat free mass (kg)	46.2 ± 4.2		47.4 ± 5.9		0.529
Smoking	3 (27)		4 (15)		0.390^b^
ACR 2016 diagnosis	10 (91)		24 (89)		1.000^b^
FIQ	50 ± 11		39 ± 16	1	0.043*
PCS	14 [11–27]		15 [10–23]		0.540^a^
STAI-trait	51 ± 5		42 ± 11	1	0.012*
Pain NRS at rest	3 [2–5]		3 [2–5]	1	0.402^b^
Postexercise pain NRS	6 [3–8]		5 [3–8]	1	0.802^a^
Comorbidities					
Cardiovascular	1 (9)		2 (7)		1.000^b^
Endocrinological	0(0)		7 (26)		0.084^b^
Psychiatric	2 (18)		3 (11)		0.615^b^
Pulmonary	5 (46)		3 (11)		0.031^b^*
Neurological	5 (46)		9 (33)		0.482^b^
Total diagnoses count	2 [1–4]		2 [0–3]		0.242^a^
Medications					
Antidepressant	4 (36)		17 (63)		0.167^b^
Opioid	2 (18)		4 (15)		1.000^b^
Anticonvulsant	5 (46)		8 (30)		0.457^b^

Parametric data expressed as mean ± SD, nonparametric data as median [interquartile range] and categorical data as count (%). *P* values refer to unpaired t-test, except for a, refers to Mann–Whitney U test, and b, refers to Pearson *Χ*^*2*^ or Fisher’s Exact Test

*ACR* American College of Rheumatology, *WPI* widespread pain index, *SS* symptom severity, *FIQ* Fibromyalgia Impact Questionnaire, *PCS* Pain Catastrophizing Scale, *STAI* State-Trait Anxiety Inventory, *NRS* numeric rating scale

^*^P < 0.05

^a^Mann-Whitney U Test

^b^Pearson *Χ*^*2*^ or Fishers's Exact Test

## Discussion

### Main results

The 29% lower $\dot{\text{V}}$O_2peak_ (mL∙min^-^^1^∙kg^-^^1^) in FM patients in this study is in concordance with previous studies where a cycle ergometer exercise was used [41–43]. The between-group difference in $\dot{\text{V}}$O_2peak_ did not dissipate when adjusted for FFM, demonstrating that lower $\dot{\text{V}}$O_2peak_ in the FM group was not related to body composition. In healthy fit subjects, $\dot{\text{V}}$O_2peak_ is limited primarily by $\dot{\text{Q}}$, whereas mitochondrial oxidative capacity is the primary limiting factor in unfit subjects [44]. It should be noted that although $\dot{\text{Q}}$ and C(a-v)O_2_ are separate factors in the Fick principle, there is interdependence between the two variables [45]. In this study, both central ($\dot{\text{Q}}$ 12% and $\dot{\text{Q}}$ _i_ 6% lower in the FM group) and peripheral (C(a-v)O_2_ 13% lower in the FM group) mechanisms contributed to lower $\dot{\text{V}}$O_2peak_ in FM, although the difference in peak $\dot{\text{Q}}$ _i_ was not statistically significant. $\dot{\text{Q}}$ (a product of HR and SV), in turn, was limited by HR_peak_, while SV was similar between groups. Lower HR_peak_ is a common finding in FM exercise studies [41, 42, 46–48]. In addition to submaximal effort, lower HR has been proposed to be a consequence of metabolic impairment and dysregulation of the autonomic nervous system [42, 47, 48]. We examined the associations between HR_peak_ respectively with pain ratings, LTPA, and symptom severity in the FM group, but we did not find any correlations. $\dot{\text{V}}$O_2peak_ and P_peak_, however, were negatively associated with baseline pain ratings. C(a-v)O_2_ is affected by not only oxygen extraction and muscle oxidative capacity but also vascular function and blood flow distribution. Exercise increases bloodflow to the working muscles via peripheral vasodilatation, while sufficient vascular resistance needs to be maintained to ensure adequate MAP [49]. MAP and SVR responses to incremental exercise were similar between groups (Fig. 4 B-D), indicating functioning vascular control in FM at a whole-body level. Additionally, lower C(a-v)O_2_ could be a consequence of lower mitochondrial oxidative phosphorylation and oxygen demand or lower capillary density in the exercising muscle. Muscle capillary density [50], mitochondrial function [51], and hence the capability for greater oxygen extraction are increased with exercise training, while deconditioning reduces mitochondrial enzymatic activity [51]. We did not, however, find significant correlations between LTPA and C(a-v)O_2_ when analyzing the FM group. Obesity does seem to affect C(a-v)O_2_ [52], but if this were the case, we would have expected to see a difference in C(a-v)O_2_ already at submaximal workloads. This study does not provide an explanation for the lower C(a-v)O_2_, and in clinical settings differentiating mild myopathies from deconditioning may be problematic [18]. However, given the similar resting lactate and L/P ratio, $\dot{\text{V}}$O_2i_ at VT1, Δ$\dot{\text{V}}$O_2_/ΔP, peak $\dot{\text{V}}$E/$\dot{\text{V}}$O_2_, and RER between groups and the lower peak lactate and peak L/P ratio in the FM group, our data do not suggest an impairment in muscle metabolism.

Participants in the FM group reported low moderate to heavy LTPA and failed to meet the WHO physical activity recommendation of 150 to 300 min of weekly moderate exercise [53]. A recent study [54] in a Swiss population demonstrated positive associations of moderate and vigorous, but not light, physical activity with $\dot{\text{V}}$O_2peak_. Correlation analysis in our study yielded similar results, suggesting that low moderate to heavy LTPA is a plausible explanation for the lower $\dot{\text{V}}$O_2peak_ in the FM group. 

A few previous studies have reported exercise thresholds (ventilatory or lactate) in FM patients [5, 41, 43, 46]. Regardless of the definition and method used, they show consistently that FM patients reach these thresholds at lower V̇O_2_ and work rate. Paradoxical to the fact that exercise training shifts VTs closer to $\dot{\text{V}}$O_2peak_ [55], VT1% and VT2% in our study were higher in the FM group, while VTs in absolute terms were lower. This is consistent with the study by Valim et al. [46]. Higher relative VTs could be explained by submaximal exercise effort, which is supported by the notion that HR_peak_ in relation to predicted maximal HR was lower and peak BR higher in the FM group. Submaximal effort of FM patients has been reported earlier [42, 43, 46].

The concept of maximal effort and the issue of possible submaximal effort in the FM group needs to be addressed. Maximal oxygen uptake ($\dot{\text{V}}$O_2max_) is an important measure of cardiorespiratory fitness representing maximal level of oxidative metabolism. A plateau in $\dot{\text{V}}$O_2_ occurs near maximal exercise and this is traditionally considered to be the best evidence of achieved $\dot{\text{V}}$O_2max_ [56]. However, a clear plateau is often not achieved [57], and $\dot{\text{V}}$O_2peak_ is used instead of $\dot{\text{V}}$O_2max_. In case a $\dot{\text{V}}$O_2_ plateau is not attained, secondary criteria are used to determine maximal effort. These criteria most commonly include one or more of the following: HR ≤ 10 - 15 bpm or ≤ 5 - 10% of the age-predicted (220-age) maximum, blood lactate concentration ≥ 8 mM, or RER ≥ 1.00, 1.05, 1.10, or 1.13 [57].

Although low HR_peak_ in the FM group points towards a less than maximal effort, we argue that our peak exercise comparisons are justified. First, peak mean RER and RPE between FM and control groups were alike, indicating similar maximal effort. Second, the median postexercise lactate was ≥ 8 mM in both groups. Third, the between-group differences in peak exercise responses did not substantially change even when those not reaching maximal effort were excluded from the analysis (Table 2). Furthermore, even though we used relatively strict RER-criteria, defining maximal exercise effort using secondary criteria (including RER and HR) is ambiguous [58]. The exercise responses recorded in this study do not represent their theoretical maximum but rather demonstrate the highest achievable response in the existing circumstances, *i.e.,* peak values.

The FM patients had a pronounced, albeit not significantly different, circulatory response to increasing oxygen demand, which manifested as steeper ΔHR/Δ$\dot{\text{V}}$O_2_ (similarly to ref. [42]) and Δ$\dot{\text{Q}}$/Δ$\dot{\text{V}}$O_2_ slopes. $\dot{\text{Q}}$ is increased approximately five liters per increased liter of $\dot{\text{V}}$O_2_ [49]. Although in this study the slope of the FM group was steeper (6.6 L blood / 1 L $\dot{\text{V}}$O_2_), the value falls within one SD of the mean of healthy subjects in the study by Beck et al. [59]. The ΔHR/Δ$\dot{\text{V}}$O_2_ slope is also well within the normal range of the recently published reference values [60]‬.‬‬‬‬‬‬‬‬‬‬‬‬‬‬‬‬‬‬‬‬‬‬‬‬‬‬‬‬‬‬‬‬‬‬‬‬‬‬‬‬‬‬‬‬‬‬‬‬‬‬‬‬‬‬‬‬‬‬‬‬‬‬‬‬‬‬‬‬‬‬‬‬‬‬‬‬‬‬‬‬‬‬‬‬‬‬‬‬‬‬‬‬‬‬‬‬‬‬‬

We noted a possible association between the ability to reach maximal effort and FIQ and STAI-trait (Table 3), although neither FIQ nor STAI-trait correlated with $\dot{\text{V}}$O_2peak_. In contrast, others have reported an association between cardiorespiratory fitness (assessed by the 6-min walk test) and STAI [61] as well as disease severity (assessed by the Revised Fibromyalgia Impact Questionnaire (FIQR) [62] in FM. As mentioned earlier, difficulties in reaching maximal effort in patients with FM has been reported before, but this has not been connected to disease severity or psychological factors. The notion that asthma, even when controlled, could affect exercise effort is not surprising considering the myriad ways, including the fear of triggering symptoms, that asthma can affect physical capacity [63]. In addition, even though the resting spirometry of the asthmatic participants was normal, we cannot rule out exercise-induced bronchial reactivity, as postexercise spirometry was not measured.

### Strengths and limitations

Although the sample size was adequate for the primary outcomes, the subgroups in our secondary analysis were small, diminishing statistical reliability. The patient and control groups were not entirely homogeneous regarding their anthropometrics, educational and employment status. This reflects real-life differences between patients with FM and their same-aged peers. Although obesity has a multitude of systemic effects, adipose tissue does not affect oxygen uptake during exercise, and $\dot{\text{V}}$O_2_ between obese and lean subjects is similar when corrected for FFM [64, 65]. Gathering LTPA data with more objective methods, such as accelerometers, would yield more reliable results. Patients with FM may be inaccurate in estimating their physical activity, but overreporting of moderate and vigorous activity is observed also in healthy individuals [9].The questionnaires were not completed at the time of the exercise test. Nevertheless, FIQ, PCS, and STAI-trait seem to be relatively stable over time [23, 66, 67]. Although studies on the PhysioFlow impedance cardiography have proven acceptable reliability in both healthy subjects and pulmonary patients and in submaximal as well as maximal exercise [34, 68, 69], other studies have shown overestimation of cardiac output in chronic obstructive pulmonary disease [70] and chronic heart failure patients [71]. Moreover, the subjects of the aforementioned studies are predominantly male, whereas participants in our study were women. As we have not measured C(a-v)O_2_ directly, but rather solved it from the Fick equation, any imprecision in measuring cardiac output would additionally impact our C(a-v)O_2_ results. The study population consisted of only women, and our results cannot be extrapolated to male FM patients.

The main strength of this study lies in the simultaneous recording of ventilatory gas exchange and ICG data. To the best of our knowledge, exercise responses in patients with FM have not been studied this intensively before.

## Conclusions

Patients with FM display poor cardiorespiratory fitness and both cardiac output and arteriovenous oxygen difference were lower compared with healthy controls. Abnormal muscle metabolism seems unlikely, whereas a possible explanation for the observed lower $\dot{\text{V}}$O_2peak_ is deconditioning and less moderate to heavy LTPA.

## Data Availability

The datasets generated and analyzed during the current study are not publicly available as consent for this was not asked from the study subjects. The data are available from the corresponding author on reasonable request if also approved by our ethics committee.

## Abbreviations

FM: Fibromyalgia
$$\dot{\text{V}}$$O_2_: Oxygen uptake
$$\dot{\text{Q}}$$: Cardiac output
C(a-v)O_2_: Arteriovenous oxygen difference
FFM: Fat-free body mass
SD: Standard deviation
$\dot{\text{V}}$O_2peak_: Peak oxygen uptake
MM: Mitochondrial myopathy
CPET: Cardiopulmonary exercise test
RER: Respiratory exchange ratio
HR: Heart rate
P: Work rate
VT: Ventilatory threshold
SV: Stroke volume
SVR: Systemic vascular resistance
$$\dot{\text{V}}$$_E_: Ventilation
$\dot{\text{V}}$CO_2_: Carbon dioxide production
LTPA: Leisure-time physical activity
ACR: American College of Rheumatology
FIQ: Fibromyalgia Impact Questionnaire
PSS: Perceived Stress Scale
STAI: State-trait Anxiety Inventory
PCS: Pain Catastrophizing Scale
WPI: Widespread Pain Index
SS: Symptom Severity
BMI: Body-mass index
RPE: Rate of perceived exertion
NRS: Numeric rating scale
L/P: Lactate-to-pyruvate ratio
VT1: First ventilatory threshold
VT2: Second ventilatory threshold
SpO_2_: Arterial oxygen saturation
ICG: Impedance cardiography
SAP: Systolic arterial blood pressure
DAP: Diastolic arterial blood pressure
MAP: Mean arterial pressure
ANOVA: Analysis of Variance
MANOVA: Multivariate analysis of variance
$\dot{\text{V}}$O_2i_: Oxygen uptake index
$\dot{\text{Q}}$_i_: Cardiac output index
SV_i_: Stroke volume index
SVR_i_: Systemic vascular resistance index
VT1%: Oxygen uptake at first ventilatory threshold as a percentage of peak oxygen uptake
VT2%: Oxygen uptake at second ventilatory threshold as a percentage of peak oxygen uptake
BR: Breathing reserve
PETO_2_: End-tidal oxygen partial pressure
PETCO_2_: End-tidal carbon dioxide partial pressure
VD/VT: Dead space to tidal volume ratio
FM_me_: Maximal effort fibromyalgia group
CTRL_me_: Maximal effort control group
WHO: World Health Organization
$\dot{\text{V}}$O_2max_: Maximal oxygen uptake
